# A correlation study between in-brace correction, compliance to spinal orthosis and health-related quality of life of patients with Adolescent Idiopathic Scoliosis

**DOI:** 10.1186/1748-7161-9-1

**Published:** 2014-02-22

**Authors:** Siu Ling Chan, Kenneth MC Cheung, Keith DK Luk, Kenneth WH Wong, Man Sang Wong

**Affiliations:** 1 The University of Hong Kong, Pokfulam, Hongkong; 2 Department of Orthopaedics and Traumatology, Queen Mary Hospital, Pok Fu Lam, Hong Kong; 3 Department of Prosthetics and Orthotics, Queen Mary Hospital, Pok Fu Lam, Hong Kong; 4 Interdisciplinary Division of Biomedical Engineering, The Hong Kong Polytechnic University, Hung Hom, Hong Kong

**Keywords:** Adolescent Idiopathic Scoliosis, In-brace correction, Compliance, Quality of life

## Abstract

**Background:**

It has been proposed that in-brace correction is the best guideline for prediction of the results of brace treatment for patients with Adolescent Idiopathic Scoliosis (AIS). However, bracing may be a stressful experience for patients and bracing non-compliance could be psychologically related. The purpose of this study was to assess the correlation between brace compliance, in-brace correction and QoL of patients with AIS.

**Methods:**

Fifty-five patients with a diagnosis of AIS were recruited. All were female and aged 10 years or above when a brace was prescribed, none had undergone prior treatment, and all had a Risser sign of 0–2 and a Cobb angle of 25-40°. The patients were examined in three consecutive visits with 4 to 6 months between each visit. The Chinese translated Trunk Appearance Perception Scale (TAPS), the Chinese translated Brace Questionnaires (BrQ) and the Chinese translated SRS-22 Questionnaires were used in the study. The in-brace Cobb angle, vertebral rotation and trunk listing were also measured. Patients’ compliance, in-brace correction and patients’ QoL were assessed. To identify the relationship among these three areas, logistic regression model and generalized linear model were used.

**Result:**

For the compliance measure, a significant difference (p = 0.008) was detected on TAPS mean score difference between Visit 1 and Visit 2 in the least compliant group (0–8 hours) and the most compliant group (17–23 hours). In addition, a significant difference (p = 0.000) was detected on BrQ mean score difference between Visit 2 and Visit 3 in the least compliant group (0–8 hours) and the most compliant group (17–23 hours). For the orthosis effectiveness measure, no significant difference was detected between the three groups of bracing hours (0–8 hours, 9–16 hours, 17–23 hours) on in-brace correction (below 40% and 40% or above). For the QoL measure, no significant difference was detected between the two different in-brace correction groups (below 40% and 40% or above) on QoL as reflected by the TAPS, BrQ and SRS-22r mean scores.

**Conclusion:**

The results showed a positive relationship between patients’ brace wear compliance and patients’ QoL. Poor compliance would cause a lower QoL.

## Background

It had been documented that some researchers believe that orthotic treatment does not alter the natural history of AIS, whereas others believe it can help stop some curves from progressing [1]. From the landmark randomized multicenter study of Weinstein et al. [2], however, it confirmed that the orthotic treatment can help to decrease the progression of curve to the threshold for surgery. Nonetheless, researchers have shown that the best guideline for predicting the results of brace treatment is the response of the spinal curve to the brace, especially during the first year of treatment. Thus, to truly reflect the effectiveness of orthotic treatment, in-brace correction should be evaluated in this respect. More importantly, patients’ compliance with orthotic treatment, i.e., brace wear compliance, should not be ignored because corrective bracing yields a favourable outcome when the patient is compliant [3]. Weiss [4], in fact, reported that a direct relationship exists between outcome and patient's compliance with orthotic treatment. Weinstein et al. [2] also detected that a significant association was found between the average hours of daily brace wear and the likelihood of a successful outcome. Landauer et al. [3] highlighted the importance of compliance: patients with good initial correction can expect a final outcome of around 7° improvement in the Cobb angle. However, bracing may be a stressful and traumatic experience, and compliance with an orthotic treatment protocol likely depends upon the patient’s physical, emotional and social well-being. Rivett et al. [5] stated that AIS may itself precipitate social problems for the patients, with orthotic treatment further affecting self- and body image, interactions with others and overall quality of life. Lack of compliance to orthotic treatment could have a psychological element.

As reflected by the previous studies, there seem to be relationships between the effectiveness of orthosis, patients’ compliance and patients’ QoL. Scientifically, a more reliable and representative outcome measure can be expected by assessing patients’ compliance on both a subjective and an objective bases. The relationship between patients’ compliance and orthosis effectiveness could be correlated, as well as the orthosis-wear behaviour as shown by the QoL measure. Therefore, there is a need to explore the correlation between the effectiveness of orthosis, patients’ compliance with spinal orthosis as assessed by subjective and objective measures and their effect on patients’ QoL. The purpose of this study was to assess the correlation between the effectiveness of orthosis in terms of in-brace correction, compliance with spinal orthosis and the HRQoL of patients with AIS during the initial treatment period.

## Methods

This was a prospective correlational study that explored the relationships between the effectiveness of orthosis (in-brace correction), compliance with orthosis and the HRQoL of the patients with AIS. SRS inclusion criteria as set by the SRS committee were adopted. Fifty five patients were recruited from the Department of Prosthetics and Orthotics at one of the local hospitals in Hong Kong. All had a diagnosis of AIS, all were female aged 10 years or above when the brace was prescribed, and none had undergone prior treatment. All had a Risser sign of 0 to 2 and a Cobb angle that measured between 25° and 40°. Approval for the study was obtained from the IRB of HKU/HA HKW.

Three areas – the effectiveness of orthosis, patients’ compliance and patients’ QoL – were assessed. The effectiveness of orthosis refers to the physical changes that result from orthotic treatment, and the focus was on in-brace correction. A TLSO (underarm brace), named the Hong Kong brace, was used for scoliotic curves at the thoracic region (with curve apex below T8) and/or lumbar region. Orthotists followed the standard procedures of casting, rectification, fabrication and fitting of the tailor-made orthosis. They adopted the three-point pressure system in making the HK brace. The brace-treated subjects were instructed to apply the correct pressure by pulling the straps of the brace to the marks on the straps that were designated by the orthotists. Clinical parameters such as Cobb angle, vertebral rotation and trunk listing were measured prospectively. Compliance refers to patient's compliance with spinal orthosis. The subjects were asked to log the time spent wearing the brace each day on the Log Sheet for Wearing Orthosis. Instruction was given to subjects and their parents on how to log the brace wearing time. In addition, they were instructed to use the remark column of the Log Sheet to log the non brace wearing time. These self-reported data were reviewed daily by the parents and at every follow-up visit by the author. Apart from the subjective log-sheet data, objective data were obtained from an orthosis monitoring system that was installed on patient’s brace. The system was used for checking patient’s compliance. Brace wearing time was recorded via a force sensor. Due to the limited supply of the orthosis monitoring systems, the tracking period for the subjects ranged only from 2 to 4 months. QoL refers to the psychosocial aspects of orthotic treatment. QoL instruments including the Chinese version of the SRS-22 outcome instrument, the Chinese version of Trunk Appearance Perception Scale and the Chinese version of Brace Questionnaire were adopted in this study. These questionnaires were chosen because of their user-friendliness, satisfactory internal consistency, reproducibility and responsiveness to change in QoL of patients with AIS treated with bracing [6,7].

Regarding the subjects, they returned to the clinic every 4 to 6 months for follow-up after their initial visit. Two consecutive follow-up examinations were performed. The physical parameters were measured at each visit. During Visit 1, subjects were asked to fill out the TAPS questionnaires. During Visits 2 and 3, subjects needed to fill out the TAPS, BrQ and SRS-22r questionnaires and to submit the daily log sheets for brace wear. To measure the correlation between orthosis effectiveness, patients’ compliance with brace wear and patients’ QoL, the in-brace correction (Cobb angle), the compliance pattern, TAPS, BrQ and SRS-22r mean scores were analysed. The relevant data from each visit were summarised by descriptive statistics, including mean, standard deviation, minimum and maximum. To identify the relationship between patients’ compliance and in-brace correction, the logistic regression model was used. To explore the relationship between patients’ compliance and patients’ QoL, the GLM was used. The GLM was also adopted to explain the relationship between in-brace correction and patients’ QoL. By analysis of the data collected by the orthosis monitoring systems, wearing time vs prescribed time was determined and reported as descriptive statistics including mean, standard deviation, and minimum and maximum. These values were analysed in relation to the subjective compliance data (number of wearing hours) as reported by the subjects.

## Results

Fifty-five subjects were recruited initially but a total of 23.7 per cent dropout was recorded. In the dropout, 6 of them had withdrawn from the study voluntarily, 5 of them had been lost to follow-up (no in-brace radiograph was recorded in the subsequent visits) and 2 of them had sought for alternative therapy. There were 42 subjects that were included for analysis. Among the subjects, 15 of them were of thoracic major curvatures and 27 were of thoracolumbar major curvatures. At inclusion, 22 subjects were in the pre-menarchal stage and 20 subjects were in the post-menarchal stage. The mean age was 12.60 years (± 1 SD: 1.01, range: 11–15), the mean menarchal status was 3.31 months (± 1 SD: 4.30, range: 0–12 months) and 26 subjects had Risser sign 0, 11 had a Risser sign 1 and 5 had a Risser sign 2. Subjects were followed up in 3 consecutive visits. The physical parameters such as Cobb angle, vertebral rotation and trunk listing were measured in each visit. The 95% confidence interval for intraobserver variability of Cobb angle, vertebral rotation and trunk listing measurement was 2.8°, 2° and 3.2 mm respectively, upon Visit 1, 3.2°, 1.8° and 4.2 mm, respectively, upon Visit 2, and 3.0°, 1.6° and 4.5 mm, respectively, upon Visit 3.

During Visit 1, the subjects filled out the TAPS questionnaires before brace fitting. During Visit 2 and Visit 3, the subjects filled out the TAPS, BrQ and SRS-22r questionnaires, submitted the daily log sheets of brace wear and underwent in-brace radiography. The average in-brace Cobb angle correction upon Visit 2 and Visit 3 was 10.9° (± 1 SD: 5.6, range: 2.5-26.0°) and 10.4° (±1 SD: 5.9, range: 0.2-28.9°) respectively. Moreover, the correction effect was higher for the thoracolumbar curve patterns (mean: 12.9°, ±1 SD: 5.69, range: 2.5-26.0°) than for the thoracic curve patterns (mean: 7.5°, ±1 SD: 3.45, range: 3.3-14.7°) upon Visit 2. The correction effect was also higher for the thoracolumbar curve patterns (mean: 12.9°, ±1 SD: 5.2, range: 3.6-28.9°) than for the thoracic curve patterns (mean: 5.9°, ±1 SD: 4.5, range: 0.2-14.7°) upon Visit 3. The mean, ±1 SD and range of physical parameters, curve patterns, bracing hours as recorded on the log sheets and the respective QoL data for the three visits were summarised in Table 1.

**Table 1:** Descriptive statistics of physical parameters, curve patterns, bracing hours and BrQ, TAPS and SRS22r scores upon the three visits (N = 42)

**Variables**	**Visit 1 (pre-brace)**	**Visit 2 (in-brace)**	**Visit 3 (in-brace)**
Cobb angle (°)	29.4 (3.9, 25.0-36.0)	18.4 (6.5, 4.2-30.4)	19.0 (6.7, 1.3-32.8)
Vertebral rotation (°)	6.8 (5.0, 0–17.5)	6.3 (5.2, 0–20)	6.4 (4.9, 0–22.5)
Trunk listing (mm)	14.60 (9.93, 0.0-39.1)	13.19 (11.17, 0.0-43.7)	11.65 (9.67, 0.0-45.1)
Thoracic curve pattern (n = 15)			
Cobb angle (°)	30.2 (4.3, 25.2-36.0)	22.7 (4.0, 15.4-30.4)	24.3 (3.9, 16.8-32.8)
In-brace correction (%)	---	18.9 (13.1, 0.7-42.5)	24.4 (10.3, 12.7-43.0)
Thoracolumbar curve pattern (n = 27)			
Cobb angle (°)	28.9 (3.6, 25.0-35.4)	16.0 (6.4, 4.2-25.8)	16.0 (6.0, 1.3-28.2)
In-brace correction (%)	---	45.0 (18.1, 14.3-95.7)	44.9 (20.0, 9.2-86.1)
Bracing hours per day (per log sheet)	---	18.31 (6.17, 0–23)	17.36 (5.98, 1–23)
TAPS (1–5)	3.56 (0.55, 2.7-4.3)	3.73 (0.56, 2.3-5.0)	3.56 (0.68, 1.7-5.0)
TAPS question 1 (1–5)	3.33 (0.79, 2–5)	3.67 (0.69, 2–5)	3.50 (0.71, 2–5)
TAPS question 2 (1–5)	3.67 (0.57, 3–5)	3.83 (0.54, 3–5)	3.69 (0.68, 2–5)
TAPS question 3 (1–5)	3.69 (0.64, 2–5)	3.69 (0.68, 2–5)	3.50 (0.77, 1–5)
BrQ total (20–100)	---	77.07 (9.41, 56.5-94.1)	76.86 (11.91, 44.1-96.5)
BrQ general health perception (1–5)	---	3.48 (0.76, 1.5-5.0)	3.30 (0.73, 1.5-5.0)
BrQ physical functioning (1–5)	---	3.73 (0.59, 2.14-4.86)	3.83 (0.68, 2.14-5.0)
BrQ emotional functioning (1–5)	---	3.61 (0.72, 2.0-5.0)	3.60 (0.79, 1.4-5.0)
BrQ self-esteem and aesthetics (1–5)	---	2.55 (0.97, 1.0-4.5)	2.50 (1.05, 1.0-5.0)
BrQ vitality (1–5)	---	2.82 (0.87, 1.0-5.0)	3.13 (0.97, 1.0-5.0)
BrQ school activity (1–5)	---	3.77 (0.60, 2.33-5.0)	3.94 (0.71, 2.33-5.0)
BrQ bodily pain (1–5)	---	4.54 (0.56, 3.14-5.0)	4.40 (0.60, 2.57-5.0)
BrQ social functioning (1–5)	---	4.16 (0.66, 2.29-5.0)	4.11 (0.8283, 1.71-5.0)
SRS22r total (22–110)	---	88.81 (10.34, 65–105)	88.79 (11.30, 52–105)
SRS22r function/activity (1–5)	---	4.34 (0.48, 2.8-5.0)	4.35 (0.56, 3.0-5.0)
SRS22r pain (1–5)	---	4.45 (0.46, 3.4-5.0)	4.49 (0.50, 3.0-5.0)
SRS22r self image/appearance (1–5)	---	3.39 (0.66, 1.6-4.6)	3.38 (0.60, 1.6-5.0)
SRS22r mental health (1–5)	---	4.04 (0.76, 2.2-5.0)	4.07 (0.81, 1.2-5.0)
SRS22r satisfaction with management (1–5)	---	3.87 (0.76, 1.5-5.0)	3.68 (0.91, 1.0-5.0)

TAPS question, BrQ & SRS22r domain scale 1 = worst, 5 = best.

### Correlation between brace compliance and in-brace correction

On Visit 2, the in-brace Cobb angle was compared to that of Visit 1 (pre-brace visit). Subjects were categorised into two groups according to in-brace correction: 40% or above (n = 13) and below 40% (n = 29). In addition, subjects were categorised according to bracing hours as follows: 0–8 hours (group 1), 9–16 hours (group 2) and 17–23 hours (group 3). Upon Visit 2, there were 4 subjects in group 1, 7 subjects in group 2 and 31 subjects in group 3. The percentages of in-brace correction (40% or above and below 40%) were entered into a logistic regression model as dependent variables. In the final model, no significant difference was detected between the three groups of bracing hours on in-brace correction. The in-brace Cobb angle upon Visit 3 was compared to that of Visit 1. Upon Visit 3, there were 18 subjects with in-brace correction 40% or above and 24 subjects with in-brace correction below 40%. Regarding bracing hours, there were 5 subjects in group 1, 8 subjects in group 2 and 29 subjects in group 3. According to the same model, no significant difference was observed between the three groups on in-brace correction. However, there was a trend that for those with shorter (0–8) bracing hours, the in-brace correction would have less than 40% (OR: 1.19, 95% C.I.: 0.080-17.71). On the other hand, a significant difference (p = 0.016) was observed between the two groups of in-brace correction on curve patterns (Table 2). Those of thoracic curves (n = 15) would have in-brace correction less than 40% (OR: 9.06, 95% C.I.: 1.51-54.47).

**Table 2:** Analysis of in-brace correction between subjects with different curve patterns adjusted for age and bracing hours (per log sheet)

**Predictor**	**OR**	**p value**	**95% C.I.**
Curve pattern			
Thoracic curves	9.06	0.016	(1.51, 54.47)
Thoraco-lumbar curves	1		
Bracing hour			
0-8	1.19	0.90	(0.08, 17.71)
9-16	0.96	0.96	(0.15, 6.13)
17-23	1		
Age	1.06	0.85	(0.58, 1.93)

Independent variables Risser sign and menarchal status were not included in the final model as they were not significant (p > 0.025).

### Correlation between patients’ compliance and patients’ quality of life

Bracing hours were categorised into three groups: 0–8 hours (group 1), 9–16 hours (group 2) and 17–23 hours (group 3). Upon Visit 2, there were 4 subjects in group 1, 7 subjects in group 2 and 31 subjects in group 3. Bracing hours (the three groups), age, Risser sign, menarchal status and curve pattern were entered into the model as predictors, and the difference in TAPS mean score between Visit 1 and Visit 2 was entered as the outcome variable. In the final model, a significant difference (p = 0.008) in the TAPS mean score difference was detected between group 1 (mean = −0.42, SD = 0.42) and group 3 (mean = 0.26, SD = 0.49) (Table 3). From the model, it was predicted that the TAPS mean score difference of group 1 (0–8 hours) was 0.70 less than that of group 3 (17–23 hours).

**Table 3:** Analysis of difference of TAPS mean score between between Visit 1 and Visit 2 on bracing hours (per log sheet) adjusted for age and curve pattern

**Predictor**	**Regression coefficient**	**p value**	**95% C.I.**
Bracing hour			
0-8	−0.70	0.008	(−1.21, -0.19)
9-16	−0.09	0.65	(−0.51, 0.32)
17-23	0		
Age	−1.25	0.22	(−0.23, 0.05)
Curve pattern	0.25	0.80	(−0.28, 0.36)

Independent variables Risser sign and menarchal status were not included in the final model as they were not significant (p > 0.025).

Upon Visit 3, there were 5 subjects in group 1, 8 subjects in group 2 and 29 subjects in group 3. Bracing hours, age and curve pattern were entered into the model as predictors and the difference in BrQ mean score between Visit 2 and Visit 3 was taken as the outcome variable. In the final model, a significant difference (p = 0.000) was detected for the difference in BrQ mean score between group 1 (mean = −12.35, SD = 12.91) and group 3 (mean = 1.70, SD = 6.94). From the model, it was estimated that BrQ mean score difference of group 1 (0–8 hours) was 16.28 less than that of group 3 (17–23 hours) (Table 4). Using the same model, significant differences were found between the groups regarding the BrQ domain mean score difference on general health perception (p = 0.002) (Table 5), physical functioning (p = 0.001) (Table 6), emotional functioning (p = 0.002) (Table 7) and bodily pain (p = 0.001) (Table 8). In addition, a significant difference was detected on BrQ bodily pain mean score difference on curve pattern (p = 0.02) (Table 8). However, no significant difference was detected for the difference of TAPS and SRS22r mean score between the two visits.

**Table 4:** Analysis of difference of BrQ mean score between Visit 2 and Visit 3 on bracing hours (per log sheet) adjusted for age and curve pattern

**Predictor**	**Regression coefficient**	**p value**	**95% C.I.**
Bracing hour			
0-8	−16.28	0.000	(−24.46, -8.10)
9-16	−2.217	0.49	(−8.61, 4.18)
17-23	0		
Age	0.31	0.78	(−1.90, 2.53)
Curve pattern	−3.78	0.17	(−9.27, 1.71)

Independent variables Risser sign and menarchal status were not included in the final model as they were not significant (p > 0.025).

**Table 5:** Analysis of difference of BrQ general health perception mean score between Visit 2 and Visit 3 on bracing hours (per log sheet) adjusted for age and curve pattern

**Predictor**	**Regression coefficient**	**p value**	**95% C.I.**
Bracing hour			
0-8	−1.27	0.002	(−2.04, -0.50)
9-16	−0.17	0.09	(−1.12, 0.08)
17-23	0		
Age	0.31	0.30	(−0.10, 0.32)
Curve pattern	−3.78	0.58	(−0.66, 0.37)

Independent variables Risser sign and menarchal status were not included in the final model as they were not significant (p > 0.025).

**Table 6:** Analysis of difference of BrQ physical functioning mean score between Visit 2 and Visit 3 on bracing hours (per log sheet) adjusted for age and curve pattern

**Predictor**	**Regression coefficient**	**p value**	**95% C.I.**
Bracing hour			
0-8	−0.80	0.001	(−1.26, -0.35)
9-16	0.09	0.63	(−0.27, 0.44)
17-23	0		
Age	0.07	0.26	(−0.05, 0.19)
Curve pattern	−0.06	0.69	(−0.37, 0.25)

Independent variables Risser sign and menarchal status were not included in the final model as they were not significant (p > 0.025).

**Table 7:** Analysis of difference of BrQ emotional functioning mean score between Visit 2 and Visit 3 on bracing hours (per log sheet) adjusted for age and curve pattern

**Predictor**	**Regression coefficient**	**p value**	**95% C.I.**
Bracing hour			
0-8	−1.08	0.002	(−1.72, -0.44)
9-16	0.16	0.52	(−0.34, 0.67)
17-23	0		
Age	−0.06	0.52	(−0.23, 0.12)
Curve pattern	−0.06	0.64	(−0.53, 0.33)

Independent variables Risser sign and menarchal status were not included in the final model as they were not significant (p > 0.025).

**Table 8:** Analysis of difference of BrQ bodily pain mean score between Visit 2 and Visit 3 on bracing hours (per log sheet) adjusted for age and curve pattern

**Predictor**	**Regression coefficient**	**p value**	**95% C.I.**
Bracing hour			
0-8	−1.07	0.001	(−1.69, -0.46)
9-16	−0.34	0.16	(−0.82, 0.14)
17-23	0		
Age	0.14	0.86	(−0.15, 0.18)
Curve pattern	−0.48	0.02	(−0.89, -0.0)

Independent variables Risser sign and menarchal sheet were not included in the final model as they were not significant (p > 0.025).

### Correlation between in-brace correction and patients’ quality of life

During Visit 2, subjects were categorised into two groups: those with in-brace correction 40% or above (n = 13) and those with in-brace correction below 40% (n = 29). The TAPS mean score between Visit 1 and Visit 2 was compared. In the model, the percentage of in-brace correction, age, Risser sign, menarchal status and curve pattern were entered as predictors, and the difference in TAPS mean score between Visit 1 and Visit 2 were entered as the outcome variable. In the final model, no significant difference was detected between the two groups on TAPS mean score difference as from the analysis.

During Visit 3, there were 18 subjects with in-brace correction 40% or above and 24 subjects with in-brace correction below 40%. The BrQ mean score between Visit 2 and Visit 3 was compared. In the model, the percentage of in-brace correction, age, Risser sign, menarchal status and curve pattern were entered as predictors, and the difference in BrQ mean score between Visit 2 and Visit 3 was entered as the outcome variable. In the final model, no significant difference was detected between the two groups on BrQ mean score difference. Neither was there a significant difference in TAPS or SRS-22r mean score between the two groups on its mean score difference.

### Correlation between curve patterns and Cobb angle

In Visit 2, subjects were categorised into two groups: those with thoracic curves (n = 15) and those with thoracolumbar curves (n = 27). The Cobb angle between Visit 1 and Visit 2 was compared. In the model, age, Risser sign, menarchal status, and curve pattern were entered as predictors. The difference in the Cobb angle between Visit 1 and Visit 2 was entered as the outcome variable. In the final model, a significant difference (p = 0.002) in the outcome variable was detected between the thoracic curve pattern (mean = −7.5, SD = 3.5) and that of thoracolumbar curve pattern (mean = −12.9, SD = 5.7) (Table 9). From the model, it was predicted that those of thoracic curve pattern would have 5.4 less in difference of Cobb angle comparing with those of thoracolumbar curve pattern.

**Table 9:** Cobb angle difference between Visit 1 (pre-brace) and Visit 2 on subjects who had different curve patterns adjusted for age and bracing hours (per log sheet)

**Predictor**	**Regression coefficient**	**p value**	**95% C.I.**
Curve pattern			
Thoracic curves	5.40	0.002	(2.14, 8.66)
Thoracolumbar curves	0		
Bracing hour	2.04	0.09	(−0.34, 4.43)
Age	0.44	0.54	(−0.99, 1.86)

Independent variables Risser sign and menarchal status were not included in the final model as they were not significant (p > 0.025).

During Visit 3, there were 15 subjects with thoracic curve pattern and 27 subjects with thoracolumbar curve pattern. The Cobb angle between Visit 1 and Visit 3 was compared. Using the same model, a significant difference (p = 0.001) was detected in the Cobb angle difference between the thoracic curve pattern (mean = −5.9, SD = 4.5) and the thoracolumbar curve pattern (mean = −12.9, SD = 5.2) (Table 10). From the model, it was predicted that those of thoracic curve pattern would have 6.45 less in difference of Cobb angle comparing with those of thoracolumbar curve pattern.

**Table 10:** Cobb angle difference between Visit 1 (pre-brace) and Visit 3 on subjects who had different curve patterns adjusted for age and bracing hours (per log sheets)

**Predictor**	**Regression coefficient**	**p value**	**95% C.I.**
Curve pattern			
Thoracic curves	6.45	0.001	(2.94, 9.97)
Thoracolumbar curves	0		
Bracing hour	−0.68	0.57	(−3.10, 1.74)
Age	0.89	0.21	(−0.52, 2.30)

Independent variables Risser sign and menarchal status were not included in the final model as they were not significant (p > 0.025).

### Correlation between subjective and objective measures of compliance

To accurately evaluate patients’ compliance with brace wear, the subjective compliance data as recorded on the log sheets and the objective compliance data as logged by the orthosis monitoring systems were analysed for 14 subjects. The recorded period was between 39 to 120 days. The mean wearing hours (per day) as recorded on the log sheets by the subjects was 10.7 (± 1 SD: 5.8, range: 0–21). The mean wearing hours (per day) as read by the systems was 10.7 (± 1 SD: 5.5, range: 2.3-19). The overall mean hours of underreporting/overreporting (wearing hours as recorded on the log sheets – wearing hours as logged by the systems) was −0.1 (± 1 SD: 3.3, range: -4-6.4). The correlation between the subjective compliance and the objective compliance was significant (r = 0.83, p = 0.000).

In addition, significant correlations were detected between the BrQ domains and the relevant SRS22r domains as in Table 11. Significant correlation was also found between in-brace Cobb angle and in-brace vertebral rotation on Visit 2 (r = 0.31, p = 0.039) as well as on Visit 3 (r = 0.33, p = 0.038).

**Table 11:** Correlation between the BrQ domains and the relevant SRS-22r domains

**BrQ domain**	**SRS22r domain**	**Pearson r**
Self-esteem and aesthetics	Self image/appearance	0.56**
	Mental health	0.55**
Social functioning	Function/Activity	0.50**
	Self image/appearance	0.43**
	Mental health	0.56**

**correlation is significant at the 0.01 level (1-tailed).

## Discussion

For measuring of Cobb angle, vertebral rotation and trunk listing, computer-assisted digital radiographic measurement is used in the authors’ institution, the Picture Archiving and Communication System (PACS). Digital imaging has many advantages in terms of convenience and the ability to adjust contrast, brightness and magnification, leading to increased accuracy of measurements in comparison to manual methods [8]. Kuklo et al. [9] commented that digital measurement improves measurement precision and shows good correlation with manual measurements for the majority of AIS parameters. Shea et al. [10] reported that the 95% confidence interval for intraobserver variability was 3.3° for manual method but 2.6° for computer-assisted measurement. Srinivasalu et al. [8] also reported the 95% confidence interval for intraobserver and interobserver variability to be 1.3° and 1.26°, respectively. More importantly, Gstoettner et al. [11] reported the main source of error to be the definition of end vertebrae. When the variability in selection of the end vertebrae was eliminated, the amount of actual error in the measurements among the examiners was relatively small [12]. The source of error may be reduced for the computer-assisted measurement because the software measures the angle automatically after drawing lines through the endplates of the end vertebrae. Besides, most studies highlighted advantages of using the torsiometer in measuring vertebral rotation. In examining interobserver and intraobserver errors, Weiss [13] reported the intraobserver error as 1° and interobserver error as 3°. Furthermore, a study by McLean et al. [14] showed that 95% of the second measurements of trunk listing by the plumb line method would be expected to be within 4 mm of the first measurement. However, no previous studies measuring trunk listing by means of PACS have been documented. In this study, the author as one examiner measured all subjects’ spine radiograph. The 95% confidence intervals for intraobserver variability in Cobb angle, vertebral rotation and trunk listing measurements were 2.8°, 2° and 3.2 mm, respectively, upon Visit 1, 3.2°, 1.8° and 4.2 mm, respectively, upon Visit 2, and 3.0°, 1.6° and 4.5 mm, respectively, upon Visit 3.

### Orthosis effectiveness measure

Landauer et al. [3] suggested that an initial correction of more than 40% and good compliance had significant effects on outcome. To explore its relationship, in-brace correction was categorised as 40% or above and below 40%; and bracing hours was also divided into three groups: from the least compliance to the most compliance, for analysis. No significant difference was detected between the three categories of bracing hours on in-brace correction, but for the least compliant group of patients, the in-brace correction tended to be less than 40%. This implies that the more compliant a patient is, the greater will be the in-brace correction. Besides, Kinel et al. [15] showed wearing the Cheneau brace a minimum of 16 hours per day, resulted in less clinical deformity than resulted from non-treatment. The study of Rahman et al. [16], demonstrated that highly compliant patients (85% compliance) showed no curve progression at the end of the treatment, whereas poorly compliant patients (62% compliance) showed curve progression of more than 6°. Although Weiss and Rigo [17] reminded that it should aim for an in-brace correction of more than 40%, not all curves can be corrected to the same extent. As Landauer et al. [3] commented, compliant patients with high initial correction can expect a final correction of around 7°, whereas compliant patients with low initial correction may not see a change in the degree of curve, and poor compliance is always associated with curve progression. While compliance is always an issue that needs to be dealt with, the study result suggested that its effect on in-brace correction that should never be underestimated. As suggested by Kim et al. [1], correction of the curve should be maximised in the brace with careful fitting and adjustment of the pads by an experienced orthotist. Best practice should aim at the best in-brace correction and at the same time the best possible comfort for the patient to foster compliance. The better the in-brace correction, the better the end result. In fact, in-brace correction is negatively related to curve magnitude [18]. Poor results can be due to poor bracing and this could be verified through in-brace radiographs to assess the obtained correction. Poor results can also be due to improper management of the patient, a factor that can ultimately influence compliance [19]. With close monitoring of the patient’s compliance and in-brace correction, a successful treatment outcome can be attained.

A significant difference in in-brace correction was observed between patients with different curve patterns. It was predicted that those with thoracic curve patterns would reach in-brace correction of less than 40%. The correction effect, as reflected by the improvement in Cobb angle, was also shown to be better for the thoracolumbar curve patterns in comparison to the thoracic curve patterns, as detected in Visits 2 and 3. These findings were consistent with those reported by Weiss et al. [20] that the correction effect was highest for the lumbar and thoracolumbar curve patterns. It was explained that thoracic curve is difficult to correct in comparison to thoracolumbar curve as a result of the anatomical structure where it articulates with the ribs to form the rigid rib cage.

Previous studies have shown that the evaluation of orthosis effectiveness has mainly focused on the correction of Cobb angle after treatment. In this study, other relevant parameters such as vertebral rotation and trunk listing were measured. As mentioned by Perdriolle et al. [21], other than Cobb angle, measurement of vertebral rotation is also significant in the prognosis and treatment of scoliosis curves. The study revealed a significant positive correlation between in-brace Cobb angle and in-brace vertebral rotation in Visit 2 as well as Visit 3. This finding was consistent with the comment made by Leathermann and Dickson [22] that vertebral rotation increases with increases in Cobb angle and is a reflection of the severity of the deformity.

### Compliance measure

Regarding bracing compliance, the subjects were divided into three groups for analysis: group 1 (0–8 hours), group 2 (9–16 hours) and group 3 (17–23 hours) in this study. This was consistent with the design of the log sheet. The subjects logged their brace wearing time as 0000–0800 hours, 0800–1600 hours, or 1600–2400 hours. For a comprehensive review, subjects were asked to note the hours they did not wear the brace, such as while bathing and exercising. From the log sheets, it was not difficult to observe the brace wearing patterns of the subjects and therefore their bracing compliance. For example, those belonged to group 1 most often wore their brace at nighttime. These subjects explained that they tried to avoid the brace during the daytime because they did not want their classmates or friends know that they needed to wear a brace. Hence, they could wear the brace only when they were at home or sleeping. This showed a deep psychological effect as reflected by the poorer QoL score.

It was predicted that the difference in TAPS mean score between Visit 1 (pre-brace) and Visit 2 of the least compliant group (0–8 hours) would be significantly (−0.70) less than that of the most compliant group (17–23 hours). The effect of brace wear was obvious. It may be that patients in the least compliant group were aware they were not compliant and therefore perceived their trunk as rather deformed by the time of Visit 2. To the contrary, the patients in the most compliant group might have reckoned that they were compliant and so perceived the appearance of their trunks as improved by the time of Visit 2. This implies that the patients more or less believed in the effect of brace wear. As highlighted by Borders [23], one of the important factors that predicts compliance is the belief patient has in the treatment outcome. However, it must be noted that although the least compliant group might have believed in the effect of brace wear, they did not comply with it. Some psychosocial issues may be hidden behind this behaviour and they need to be revealed. As noted by Hawes [24], psychological issues have previously been shown to influence compliance in brace wear. The perception of body image and that of the trunk deformity are complementary [25]. Self image is decreased during the brace wearing period; however it returns to normal after completion of the brace wearing period [26]. Nevertheless, the relationship between the compliance measure and the difference in TAPS mean score between Visit 2 and Visit 3 was shown not to be significant. This may have occurred because the patients perceived the effect of brace wear had already occurred by Visit 2 and thus it made no difference to their trunk perception by the time of Visit 3, regardless of their compliance.

The study results also predicted that the difference in BrQ mean score between Visit 2 and Visit 3 of the least compliant group (0–8 hours) would be significantly (−16.28) less than that of the most compliant group (17–23 hours). Moreover, a similar value was detected for the difference in the BrQ domain mean score, which included general health perception, physical functioning, emotional functioning as well as bodily pain. Thus, it appeared that the poorer compliance with brace wear, the poorer the QoL. This result contradicted the finding of Ugwonali et al. [27], who reported that brace wear did not decrease the QoL of adolescents with AIS. However, the questionnaire that Ugwonali et al. used was not condition-specific [5]. Feise et al. [28] emphasised that disease-specific instruments are considered superior for measurements in homogenous populations because they concentrate primarily on the most significant domains of the disease and are more sensitive for measuring clinically important differences. In this study, the BrQ was adopted as a brace-oriented instrument. The questionnaire was translated into Chinese according to the guidelines of cross-cultural adaptation process used by the AAOS Outcomes Committee [29]. It was then validated by the authors. The results showed that the Cronbach’s alpha and the intra-class correlation coefficient were both 0.93, and no floor or ceiling effect was demonstrated in all the BrQ domains except the bodily pain domain that showed a ceiling effect of 37.3%. Nevertheless, the result of this correlation study was in fact consistent with the finding of Rivett et al. [5] that poor compliance to a brace protocol is associated with poorer QoL. In the present study, in comparison to the most compliant subjects, the least compliant subjects tended to have a poorer perception of their general health and function more poorly both physically and emotionally. They also tended to have more bodily pain.

Regarding bracing hours, no significant difference was detected in the BrQ physical functioning and emotional functioning mean score difference between the moderately compliant group (9–16 hours) and the most compliant group (17–23 hours). However, in the most compliant group, the mean score difference tended to be less than that of the moderately compliant group. This is in line with the comment by Edgar [30], who reported 16 hours per day as the optimum wearing time to ensure a balance between the effectiveness of and tolerance to brace wear. In the study of Weinstein et al. [2], it also highlighted brace wear for an average of at least 12.9 hours per day was associated with success rates of 90 to 93%.

Regarding use of the orthosis monitoring system, it reliably generated objective data pertaining to patients’ compliance with bracing as shown in the present study. The mean wearing hours as recorded on the log sheets by the subjects was comparable to the mean wearing hours as read by the systems. No underreporting or overreporting of the wearing hours was observed. A significant correlation was also shown between the subjective compliance (wearing hours recorded on the log sheet) and the objective compliance (wearing hours recorded by the orthosis monitoring system). Hence, the system helps to prove if there is any overestimated duration of brace wear as recorded on the self-reporting inventory. Ultimately, such data gathering will help in establishing evidence-based AIS management.

Patient’s compliance with brace wear should not be undermined, as corrective bracing has shown favourable outcomes when the patient is compliant (3). Weinstein et al. [2] also supported that bracing significantly decreased the progression of high-risk curves to the threshold for surgery in patients with AIS. It also stressed longer hours of brace wear were associated with greater benefit as shown by the dose–response relationship. To enhance patient’s compliance, the orthotists at the local hospital adopted different strategies. One was making a hard copy of the immediate in-brace radiograph and presenting it to patient. The orthotists explained to the patient how the brace helped to control the curve progression. Upon each visit, checking on patient’s compliance was done by reviewing the wear and tear of the brace and the brace strapping, patient’s skill in wearing and removing the brace, and any skin discolouration on the pressure area of the patient as created by the brace. Ways to maximise the brace tolerability and reduce visibility were also introduced, such as changing to a new brace when the patient has grown significantly and making suggestions for wearing special clothing. In this study, the author, who had regular contact with the subjects, also checked the brace and the compliance records regularly. Otherwise, appropriate counselling would be provided to the patients after assessment of their emotional status. In fact, maintaining compliance is not viewed as the sole responsibility of the patients and their families. The responsibility also lies with the treating team, which may include orthopaedic surgeons, clinical psychologists, nurses, orthotists and physiotherapists. Their roles should be highlighted in terms of enhancing patient’s compliance. As recommended by the International Scientific Society on Scoliosis Orthopaedic and Rehabilitation Team (SOSORT), there is a need for a multiprofessional expert team to effectively treat the patients through increased compliance [31].

### Quality of life measure

No relationship was found in the current study between in-brace correction and QoL. This differed from the finding of Vasiliadis et al. [7], who showed BrQ score to be related to the degree of deformity. The finding of present study suggested that QoL issues may be related more to psychosocial coping mechanisms than to physical deformity and its consequences [5]. Freidee et al. [32] noted that patients with scoliosis also reported more physical complaints independent of the seriousness of the impairment, such as the magnitude of Cobb angle. Scoliosis causes psychological distress, regardless of the severity of curve [32]. It is also true that successful treatment to prevent the progression of the curvature does not necessarily mean improvement in the QoL.

The present study showed that the brace affected QoL negatively and thus further reinforced the need to garner support from the multidisciplinary team for patients with AIS at the early stage of bracing. The ultimate purpose would be to manage patients’ psychosocial issues as provoked by the brace treatment. Reichel et al. [33] recognised that support for patients in the form of psychological group or individual sessions can help to prevent psychosocial impairment and it should be included in holistic management plans.

In exploring the relationship between self image and social functioning, a positive correlation was demonstrated by this study. As Deviren et al. [34] found, psychosocial and body image disturbances were less marked in patients with good social or family functioning. Again, it was shown to be important to provide support to patients and their families to enhance patients' QoL as well as patients' compliance with the treatment.

In this study, the BrQ and TAPS were shown to be effective in evaluating the QoL of patients with AIS. Both BrQ and TAPS exhibited superiority over the SRS-22r in detecting changes in QoL according to brace compliance. As Aulisa et al. [35] explained, this might be related to the greater number of questions contained in the BrQ that may allow it to explore more domains than the other questionnaires explore.

## Limitation

Within the study period, due to a decrease in eligible patients, the recruitment was found difficult, and the recruitment period was extended to 1 year. To allow for sensible comparison, subjects’ data were collected only during Visit 1 (pre-brace), Visit 2 and Visit 3. In these three consecutive visits, the detection of change in the outcome measures, i.e., in-brace correction, bracing compliance and QoL may not have been comprehensive. For a comprehensive understanding of the correlation between the outcome measures, it was suggested that the subjects be followed up till the completion of their treatment and then for 2 years after maturity. More importantly, it has to incorporate the SRS outcome criteria, i.e., the percentage of patients with ≦5° curve progression and the percentage of patients with ≧6° progression at maturity; the percentage of patients with curves exceeding 45° at maturity and the percentage who have had surgery recommended/undertaken; and 2-year follow up beyond maturity to determine the percentage of patients who subsequently require surgery [36].

Regarding the data collection, most of the relevant parameters were collected for analysis. While regression model was frequently used for analysis, it would be more detailed if the demographic data such as family background, educational level, household income and marital status of parents and child–parent relationship could be included as well. They may be the important factors that help to predict the outcome more substantially. For example, child–parent relationship may be a significant factor for predicting self-esteem and social functioning in BrQ.

About the data analysis, in-brace correction was categorized into two groups: 40% or above and below 40%. This was in accordance with the one suggested by Landauer (3) as high initial correction of more than 40% and good compliance were of significant impact for the outcome. It helped to predict the treatment outcome effectiveness. In a study by Knott et al. [37], however, it recommended that an effective brace should be able to achieve 50% correction of the curve magnitude, immediately after application. It was one of the SOSORT initiatives. In fact, in-brace correction for more than 50% should be the treatment goal for each patient. As highlighted by Knott et al. [37], one must first be able to distinguish effective from ineffective bracing, as there is no reason to evaluate the outcome of ineffective braces. Nonetheless, it must be emphasized that the reduction of curvature also depends on various factors such as curvature flexibility and the fitting accuracy of spinal brace.

In the compliance measure, subjective data were collected. Subjects and their parents were instructed on the use of a specially designed “Log Sheet of Wearing Orthosis”. Upon each visit, subjects had to submit the log sheets for record and analysis. In addition, checking on patients’ compliance was implemented by observing the wear and tear of the brace and the brace strapping. However, it was reported that on average patients had overreported their hours of brace wear to their physician [38]. To verify this, objective measurement of subjects’ compliance to brace wear was done by using the orthosis monitoring system in the last few months of the study. It would be most representative if the monitoring system could be applied throughout the treatment period. In so doing, the relationship between in-brace correction, bracing compliance and QoL could be evaluated in a more comprehensive and evidence-based perspective. After all, future research is required to address the optimal bracing hours that could assure an effective bracing through the monitoring system.

During the study period, in-brace radiography was not prescribed for each individual subject at each visit despite the reminder notes that were attached to the subjects’ medical records. This contributed to the high dropout rate (5 out of 55 subjects). From the medical record, it revealed that the physicians tended to measure out-brace Cobb angle to monitor any curve progression from time to time. To enhance consistency of management, it was recommended that subjects should be under the care of one physician if possible. A pre-meeting and regular meeting with the physician as well as other team members should also be organised.

## Conclusions

Literatures have suggested that QoL should be carefully monitored over the course of treatment, highlighting the difficulties patients experience when subjected to conservative treatment. In the present study, patients’ compliance patterns were observed and the effect of compliance on patients’ QoL was thus explained. The results of the study could facilitate clinicians to make adjustments to patients’ care regimens that are based upon factors that affect outcomes. Such factors include patients’ compliance, which is as important to QoL as it is to brace success. The ultimate treatment effect (in both physical and psychological aspects) can then be enhanced.

## Availability of supporting data

The data set supporting the results of this article is included within the article.

## Abbreviations

AIS: Adolescent idiopathic scoliosis; AAOS: American Academy of Orthopedic Surgeons; BrQ: Brace Questionnaire; GLM: Generalised linear model; HRQoL: Health-related quality of life; QoL: Quality of life; SOSORT: Society on Scoliosis Orthopaedic and Rehabilitation Treatment; SRS: Scoliosis Research Society; TAPS: Trunk Appearance Perception Scale; TLSO: Thoracic-lumbar-sacral orthosis.

## Competing interests

The authors declare that they have no competing interests.

## Authors’ contributions

The study described was a multidisciplinary project that involved the collaboration of health professionals from different disciplines. Ms PC coordinated and implemented the study with the team members. She involved in translating and validating the BrQ and TAPS, preparing a Chinese version the SRS-22, ensuring the readiness of the orthosis monitoring systems, collecting clinical data (recruiting subjects, distributing and collecting relevant questionnaires at every visit, measuring clinical parameters, collecting compliance data as recorded in the log sheet, installing orthosis monitoring systems, downloading compliance data from the orthosis monitoring systems, analysing the data, holding regular project reviews with the project team members, preparing reports and disseminating the finding. Dr MSW, Professor KMCC, Professor KDKL, and Mr KWHW provided support to the study by giving advice and revised the manuscript. All authors read and approved the final manuscript.

## Authors’ information

SL Chan, RN, BHSc, MPHC, Lecturer, School of Nursing, The University of Hong Kong.

KMC Cheung, MD, Professor and Head, Department of Orthopaedics and Traumatology, Queen Mary Hospital, Hong Kong.

KDK Luk, MD, Clinical Professor, Department of Orthopaedics and Traumatology, Queen Mary Hospital, Hong Kong.

KWH Wong, Professional Diploma of Prosthetics and Orthotics, Head of Department of Prosthetics and Orthotics, Queen Mary Hospital, Hong Kong.

MS Wong, Associate Professor, Interdisciplinary Division of Biomedical Engineering, The Hong Polytechnic University, Hong Kong.
